# Effects of microbial biocontrol agents on tea plantation microecology and tea plant metabolism: a review

**DOI:** 10.3389/fpls.2024.1492424

**Published:** 2025-01-20

**Authors:** Yixin Xie, Chunxia Cao, Daye Huang, Yan Gong, Beibei Wang

**Affiliations:** ^1^ National Biopesticide Engineering Research Centre, Hubei Biopesticide Engineering Research Centre, Hubei Academy of Agricultural Sciences, Wuhan, China; ^2^ College of Food Science and Technology, Huazhong Agricultural University, Wuhan, China

**Keywords:** tea, biotic stress, biological control, metabolomics, tea quality

## Abstract

The quality of fresh tea leaves is crucial to the final product, and maintaining microbial stability in tea plantations is essential for optimal plant growth. Unique microbial communities play a critical role in shaping tea flavor and enhancing plant resilience against biotic stressors. Tea production is frequently challenged by pests and diseases, which can compromise both yield and quality. While biotic stress generally has detrimental effects on plants, it also activates defense metabolic pathways, leading to shifts in microbial communities. Microbial biocontrol agents (MBCAs), including entomopathogenic and antagonistic microorganisms, present a promising alternative to synthetic pesticides for mitigating these stresses. In addition to controlling pests and diseases, MBCAs can influence the composition of tea plant microbial communities, potentially enhancing plant health and resilience. However, despite significant advances in laboratory research, the field-level impacts of MBCAs on tea plant microecology remain insufficiently explored. This review provides insights into the interactions among tea plants, insects, and microorganisms, offering strategies to improve pest and disease management in tea plantations.

## 1 Introduction

Tea is one of the three major non-alcoholic beverages in the world. Global tea demand has increased significantly during the COVID-19 pandemic (Castellana et al., 2021), a trend that may be closely related to the potential benefits of bioactive substances in tea (Gilbert, 2019; Bag et al., 2022b). Reports from the International Tea Committee (ITC) and the Food and Agriculture Organization of the United Nations (FAO) indicated that in 2022, the global annual value of tea production exceeded $17 billion, with the world tea trade amounting to approximately $9.5 billion (FAO, 2022). The tea industry is among the key agricultural activities in many countries, which is vital in sustaining rural economies (Jayasinghe and Kumar, 2021; Bermúdez et al., 2024). To ensure the production of high-quality tea, tea farmers and researchers have long been committed to these pursuits. Tea production primarily relies on tea trees (*Camellia sinensis*), and the quality of fresh tea leaves forms the foundation for high-quality finished tea. The growth conditions of tea plants, including growth environment, soil quality, climate, and cultivation practices, significantly impact the quality of fresh tea leaves. However, during the growth process, tea plants frequently face threats from pests and diseases.

Biotic stress factors such as tea blister blight (caused by *Exobasidium vexans* Massee), tea anthracnose (caused by *Colletotrichum* spp.), tea looper (*Ectropis oblique*), and tea red spider mite (*Oligonychus coffeae*) have long threatened tea yield and quality. If not effectively controlled, these pests and diseases can lead to production losses of up to 55% (Hazarika et al., 2009). Although precise data on economic losses is difficult to determine, it is estimated that these impacts may amount to billions of dollars annually. In recent years, climate change has increased the risk of new pathogens and pests in tea plantations. Rising temperatures, altered rainfall patterns, and extreme weather have expanded the range of these threats, enabling them to thrive in previously unsuitable areas (Pandey et al., 2021b). Additionally, Biotic stress activates the expression of the defense genes in tea plants such as caffeine, catechins, L-theanine, and volatile compounds (Zeng et al., 2024), thereby impacting its flavor and health benefits (Berg and Smalla, 2009). Traditionally, chemical pesticides have long been the primary method for controlling these pests and diseases in tea plantations. However, many studies have demonstrated that the application of synthetic pesticides directly affects environmental microorganisms (Wang and Cernava, 2020; Win et al., 2021) and may also indirectly influence them by interfering with plant metabolic pathways (Liu et al., 2018). For instance, glyphosate can severely weaken the defense mechanisms of glyphosate-sensitive plants (including tea plants) against microbial diseases by disrupting the shikimate pathway, thereby significantly enhancing its efficacy as an herbicide (Martinez et al., 2018).

In contrast, microbial control methods have increasingly shown advantages in tea plantations (Ahirwar et al., 2020; Idris et al., 2020). Microbial biocontrol agents (MBCAs) primarily consist of entomopathogenic and antagonistic microorganisms, which specifically target pests and pathogens while minimizing harm to non-target organisms and the environment (Roy and Muraleedharan, 2014). Compared to chemical pesticides, MBCAs offer safer and more sustainable pest management solutions. However, since MBCAs contain large quantities of exogenous microorganisms, their impact extends beyond just pest and disease control, influencing the broader tea plant ecosystem (Grosch et al., 2012). Microorganisms play a crucial role in tea plant growth by forming symbiotic relationships within and on plant surfaces, contributing to a balanced and dynamic ecosystem (Trivedi et al., 2020; Tan et al., 2022; Jibola-Shittu et al., 2024). These microbial communities primarily influence the plant’s metabolism or produce bioactive compounds, affecting the plant’s characteristic metabolites (Liao et al., 2022; Wu et al., 2024a). These interactions shape the community structure and succession of tea plant microorganisms, which are intricately linked to plant health, resilience, and overall growth (Hu et al., 2018; Xie et al., 2020; Hazarika et al., 2021; Bag et al., 2022a). For example, Xin et al (Xin et al., 2024)identified specific microorganisms in the root microbiomes of high-theanine and low-theanine tea varieties that may regulate theanine levels by influencing nitrogen metabolism. Similarly, Sun et al (Sun et al., 2019). isolated an endophytic bacterium (*Luteibacter* spp.) from tea seedlings, which exhibited biocatalytic activity by converting glutamine and ethylamine into theanine. The stability of these microbial communities is affected by various factors (Gong et al., 2024), such as pesticide application (Duke, 2018; Hage-Ahmed et al., 2019), biotic stress (Huang et al., 2023), abiotic stress (Zeng et al., 2019a; Bora et al., 2022b), etc. MBCAs act directly on the microbial community within the tea plant ecosystem and may indirectly influence tea quality by altering the structure of the microbial community (Cernava et al., 2019). Bora et al (Bora et al., 2022a). found that using microbial consortia to combat grey blight disease (caused by *Pseudopestalotiopsis curvatispora* Petch) not only suppressed the pathogens effectively but also increased rhizosphere microbial diversity and enhanced leaf nutrient content. Certain MBCAs may also induce systemic resistance in tea plants and improve their nutritional status as biofertilizers. Nevertheless, current research on commercially MBCAs has predominantly focused on the initial screening of biocontrol strains, with relatively less attention given to their practical impacts on ecological microorganisms and tea plant metabolism.

**Figure 1:**
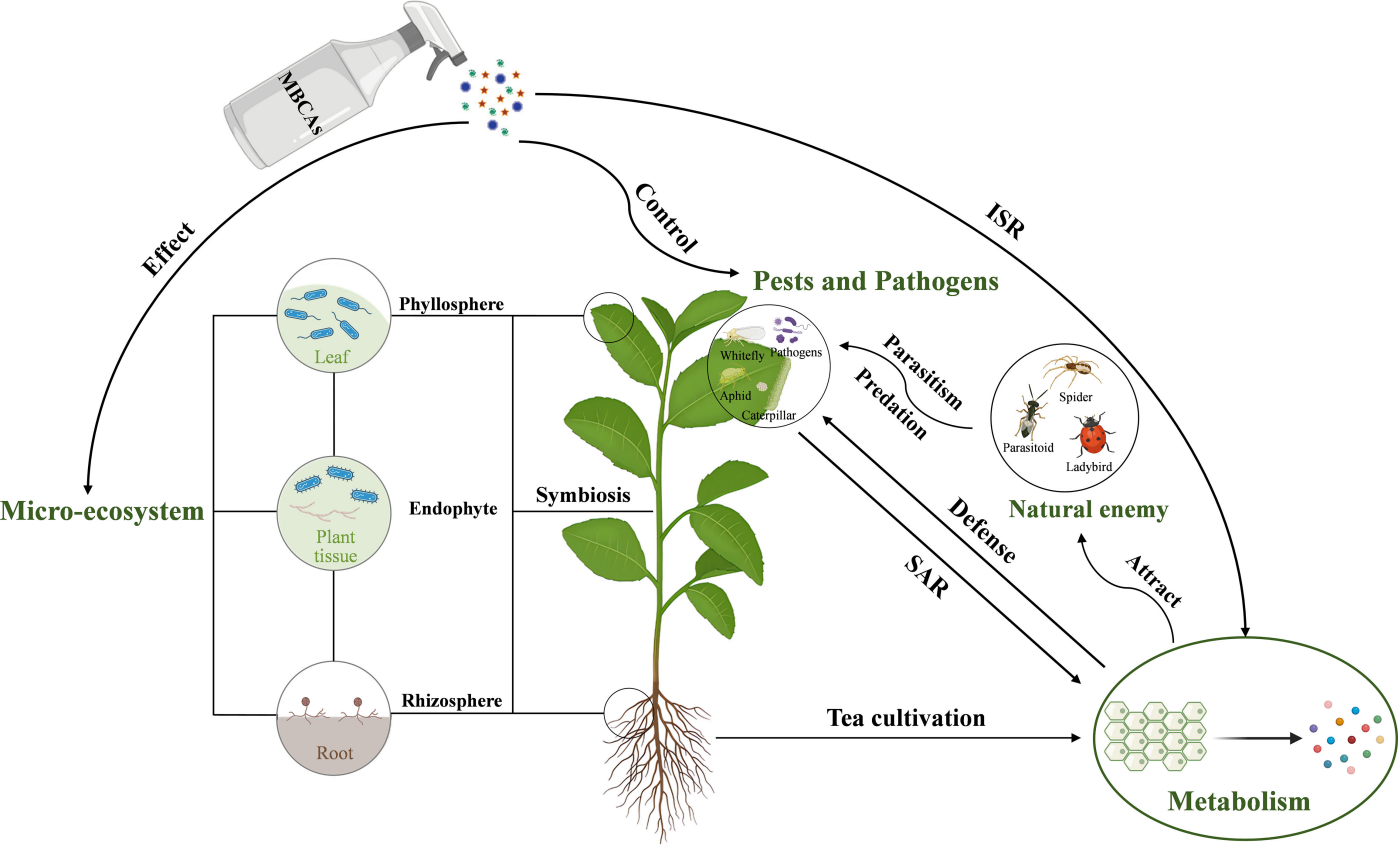
The interactions between MBCAs, tea plant microorganisms, insects, tea plant pathogens, and tea plant metabolism. MBCAs, microbial biocontrol agents; ISR, induced systemic resistance; SAR, systemic acquired resistance.

In light of these considerations, this review aims to provide a concise overview of the main types and mechanisms of commercially available or promising MBCAs used in tea plantations, their colonization efficacy, and their impact on the tea plant’s microbial community. Additionally, this review explores the influence of biotic stress and exogenous microorganisms on tea plant metabolism, while briefly introducing newly discovered microorganisms and their potential applications in the outlook section. The interactions between the main factors discussed in this review are illustrated in **Figure 1**. By offering new insights into the interactions among tea plants, insects, and microorganisms, this review aims to advance practical pest and disease management strategies in tea plantations and support the biocontrol agent industry.

## 2 Types and mechanisms of microbial biocontrol agents applied in pests and diseases control of tea

To understand the impact of MBCAs on the micro-ecosystem of tea plantations and the metabolism of tea plants, it is essential first to identify the types of biocontrol agents used and their underlying mechanisms. Some microbial resources discovered in tea plantations exhibit specific activity against pests and pathogens through unique mechanisms, highlighting their significant potential for development. Therefore, in addition to presenting MBCAs that are already widely commercialized in tea plantations, this review also introduces promising biocontrol agents with potential for future application. The MBCAs targeting major pests and pathogens in tea plantations are listed in **Tables 1**, **2**, respectively.

**Table 1:** Microorganisms with biocontrol effects on major tea pests.

Target Pest	Microbial Classification	Microbial Species/Strain
*Ectropis obliqua*	Bacteria	Bacillus thuringiensis (Barthakur, 2011)
	Fungi	*Metarhizium anisopliae* (Zhao et al., 2023a)
	Virus	Ectropis obliqua nuclear polyhedrosis virus (Ye et al., 2014)
*Caloptilia theivora*	Bacteria	*Bacillus thuringiensis* (Barthakur, 2011), *Enterobactor* sp (De et al., 2008)
*Scirtothrips dorsalis*	Bacteria	*Bacillus thuringiensis* (Gurusubramanian et al., 2008)
*Eterusia magnifica*	Bacteria	*Bacillus thuringiensis* (Mukhopadhyay et al., 2010)
*Microtermes obesi*	Bacteria	*Bacillus thuringiensis* (Singha et al., 2010)
	Fungi	*Beauveria bassiana* (Singha et al., 2011)
*Paralepidosaphes tubulorum* Ferris	Bacteria	*Serratia marcescens* (Ye et al., 2014)
*Chrysomphalus ficus* L	Bacteria	*Serratia marcescens* (Ye et al., 2014)
*Oligonychus coffeae*	Bacteria	*Pseudomonas fluorescens* (Roobak Kumar et al., 2011), *Bacillus velezensis* (Bora et al., 2023)
	Fungi	*Metarhizium anisopliae* (Deka et al., 2022a)
*Buzura suppressaria*	Fungi	*Beauveria bassiana* (Ghatak and Reza, 2007)
*Empoasca vitis*	Fungi	*Beauveria bassiana* (Feng et al., 2004)
*Myllocerinus aurolineatus*	Fungi	*Beauveria bassiana* (Pandey et al., 2021a)
*Helopeltis theivora* Waterhouse	Fungi	*Beauveria bassiana* (Deka et al., 2021)
*Aleurocanthus camphalus*	Fungi	*Paecilomyces cinnamomeus* (Saito et al., 2012)
*Microcerotermes beesoni* Snyder	Fungi	*Metarhizium anisopliae* (Kumhar et al., 2020)
*Homona magnanima*	virus	Granulosis virus (Sato et al., 1986)
*Adoxophyes* sp.	virus	Granulosis virus (Sato et al., 1986)
*Euproctis pseudoconspersa*	virus	Euproctis pseudoconspersa nuclear polyhedrosis virus (Ye et al., 2014)
*Myllocerinus aurolineatus* Voss	Fungi	*Metarhizium pingshaense* (Fu et al., 2023)
*Hyposidra talaca* Walker	virus	Hyposidra talaca NPV (Deka et al., 2023)

**Table 2:** Microorganisms with biocontrol effects on tea plant pathogens.

Target Pathogens	Microbial Classification	Microbial Species/Strain
*Curvularia eragrostidis*	Bacteria	*Serratia marcescens* (Dhar Purkayastha et al., 2018)
*Pestalotiopsis theae*	Bacteria	*Serratia marcescens* (Dhar Purkayastha et al., 2018), *Paecilomyces lilacinus* (Xu et al., 2023a), *Bacillus subtilis* (Kolandasamy et al., 2023)
	Virus	Pestalotiopsis theae chrysovirus-1 (Zhou et al., 2021)
*Colletotrichum camelliae*	Bacteria	*Serratia marcescens* (Dhar Purkayastha et al., 2018); *Bacillus velezensis* (He et al., 2024)
	Fungi	*Rhizophagus intraradices* (Chen et al., 2022)
*Lasiodiplodia theobromae*	Bacteria	*Serratia marcescens* (Dhar Purkayastha et al., 2018)
*Rhizoctonia solani*	Bacteria	*Serratia marcescens* (Dhar Purkayastha et al., 2018)
*Sphaerostilbe repens*	Bacteria	*Serratia marcescens* (Dhar Purkayastha et al., 2018)
*Fomes lamaoensis*	Bacteria	*Serratia marcescens* (Dhar Purkayastha et al., 2018), *Bacillus megaterium* (Chakraborty et al., 2006)
*Poria hypobrunae*	Bacteria	*Serratia marcescens* (Dhar Purkayastha et al., 2018)
*Ustulina zonata*	Bacteria	*Serratia marcescens* (Dhar Purkayastha et al., 2018)
*Corticium theae*	Bacteria	*Bacillus* sp (Islam et al., 2018)
	Fungi	*Aspergillus niger*, *Trichoderma atroviride, Trichoderma cithnoviride* (Thoudam and Dutta, 2012)
*Colletotrichum theae*	Bacteria	*Bacillus subtilis* (Kim et al., 2009)
*Phomopsis theae*	Bacteria	*Bacillus megaterium* (Kolandasamy et al., 2023)
	Streptomyces	*Streptomyces* sp (Marimuthu et al., 2020)
	Fungi	*Trichoderma viride* (Kolandasamy et al., 2023)
*Macrophoma theicola*	Bacteria	*Bacillus amyloliquefaciens* (Jeyaraman and Robert, 2018)
*Colletotrichum fructicola*	Bacteria	*Bacillus velezensis* (He et al., 2024)
*Colletotrichum gloeosporioides*	Bacteria	*Bacillus velezensis* (He et al., 2024), *Bacillus altitudinis* (Wu et al., 2024b)
	Fungi	*Trichoderma Asperellum* (Shang et al., 2020)
*Colletotrichum siamense*	Bacteria	*Bacillus velezensis* (He et al., 2024)
*Colletotrichum kahawae*	Bacteria	*Bacillus velezensis* (He et al., 2024)
*Exobasidium vexans*	Bacteria	*Ochrobactrum anthropi* (Sowndhararajan et al., 2013)
*Cercospora theae*	Streptomyces	*Streptomyces sannanensis* (Gnanamangai and Ponmurugan, 2012)
*Fusarium solani*	Fungi	*Trichoderma asperellum*, *Trichoderma harzianum*, *Trichoderma asperellum* (Kumhar et al., 2020); *Trichoderma reesei* (Pandey et al., 2023)
*Pseudopestalotiopsis theae*	Fungi	*Trichoderma reesei* (Pandey et al., 2022)
*Curvularia eragrostidis*	Bacteria	*Serratia marcescens* (Dhar Purkayastha et al., 2018)

### 2.1 Entomopathogenic microorganisms

Entomopathogenic microorganisms predominantly consist of bacteria, fungi, and viruses (Deka et al., 2022b). Bacterial entomopathogens primarily achieve insecticidal effects by secreting toxic proteins that inhibit pest growth (Pan et al., 2024). Research indicates that almost all insect-pathogenic bacteria isolated from tea pests belong to *Bacillus thuringiensis* (Bt) (Ye et al., 2014). The insecticidal mechanism of Bt involves the production of insecticidal crystal proteins (ICPs) within the sporangium during the sporulation phase, which exert stomach poisoning and contact toxicity effects on pests. These ICPs exhibit high specificity, becoming toxic only upon activation within specific host insects (Azizoglu et al., 2023). In the 1970s and 1980s, China employed Bt to manage *Lepidopteran* pests, achieving control efficacy rates of over 95% (Barthakur, 2011). With the continuous discovery of new strains, Bt has demonstrated effective control against other common tea plantation pests, such as tea thrips (*Scirtothrips dorsalis*) (Gurusubramanian et al., 2008), red slug caterpillar (*Eterusia magnifica*) (Mukhopadhyay et al., 2010), and tea termite (*Microterms obesi*) (Singha et al., 2010). Not only in tea plantations, but Bt has become the most widely used commercial bacterial insecticide worldwide (Bravo et al., 2011). Current research suggests that Bt’s effectiveness in tea plantation ecosystems could be further enhanced through the use of highly virulent strains (Banik et al., 2019), new delivery systems (Pan et al., 2024), or combined use with entomopathogenic fungi (Vimala Devi et al., 2020).

Apart from Bt, several other bacteria have shown potential in controlling tea plantation pests. Damayanti et al (De et al., 2008). isolated *Enterobacter* sp. from *Caloptilia theivora*, which exhibited an LC50 value of 363.1 μg/ml (bacterial weight/volume of water) against *C.theivora* larvae. Additionally, Wang et al (Ye et al., 2014). discovered that *Serratia marcescens* showed pathogenicity against two scale insects, *Paralepidosaphes tubulorum* Ferris and *Chrysomphalus ficus* (Roobak Kumar et al., 2011). Popy et al (Bora et al., 2023)found that *Pseudomonas fluorescens* and *Bacillus velezensis* exhibited lethal activity against the *Oligonychus coffeae* by secreting hydrolytic enzymes and secondary metabolites. However, most of these bacteria, aside from Bt, face significant challenges such as development difficulties, high costs, and unstable activity, which limit their extensive application in tea plantations.

Entomopathogenic fungi (EPF) thrive in ecosystems with humidity above 80%, making valuable for pest control in the hot, humid climates of tropical and subtropical tea plantations (Ye et al., 2014). The potential of *Beauveria bassiana* as a non-chemical pest control agent was first reported in 1837, leading to increased attention on EPF (Bhattacharyya et al., 2022). The main genera involved include *Beauveria*, *Metarhizium*, *Paecilomyces*, *Hirsutella*, and *Nomuraea* (Saito et al., 2012; Roy and Muraleedharan, 2014; Cheramgoi et al., 2016; Abdel-Raheem et al., 2020; Canassa et al., 2020). Notably, *B.bassiana* and *Metarhizium anisopliae* have been registered as biopesticides and are extensively employed for managing tea pests in regions including China, India, and Sri Lanka (Idris et al., 2020; McGuire and Northfield, 2020). Unlike bacteria, fungi infect insects primarily by spreading spores. The fungal hyphae penetrate the insect cuticle through mechanical pressure and the action of hydrolytic enzymes, thereby exerting a contact insecticidal effect (Wang et al., 2023; Shang et al., 2024). For example, *B.bassiana* has shown significant effectiveness in controlling various pests in tea plantations, including the tea looper (*Buzura suppressaria*) (Ghatak and Reza, 2007), false-eye leafhopper (*Empoasca vitis*) (Feng et al., 2004), tea weevil (*Myllocerinus aurolineatus*) (Pandey et al., 2021a), wood-eating tea termite (*Microtermes obesi*) (Singha et al., 2011), and tea mosquito bug (*Helopeltis theivora*) (Deka et al., 2021). Additionally, a study conducted in Japan demonstrated that a concentration of 1×10^7^ conidia/ml of *Paecilomyces cinnamomeus* led to a 90% infection rate of whiteflies (*Aleurocanthus camphalus*), which was significantly higher than other fungal insecticides available on the market (Saito et al., 2012). The spore suspension of *M.anisopliae* can achieve a mortality rate of 78% in *O.coffeae* (Deka et al., 2022a). However, most entomopathogenic fungi have poor compatibility with chemical pesticides (Joshi et al., 2018), while rainfall can promote the extensive growth of these pathogens in insects.

Insect viruses are classified into six major categories: nuclear polyhedrosis viruses (NPV), granulosis viruses (GV), entomopoxviruses, iridoviruses, ascoviruses, and picornaviruses. As pathogenic natural enemies, these viruses exhibit a high degree of specificity towards their target pests (Sood et al., 2019). They infect hosts by penetrating the body, replicating and producing specific proteins that disrupt host cells, ultimately causing insect mortality (Payne, 1982).In the 1990s, Kagoshima Prefecture in Japan used a mixture of GV for the biological control of pests *Homona magnanima* and *Adoxophyes* sp., achieving pest mortality rates of 60-75% (Sato et al., 1986). To date, 82 viruses associated with tea plant insects have been identified and reported in China. Among these, the Ectropis obliqua nuclear polyhedrosis virus (EcobNPV) and Euproctis pseudoconspersa nuclear polyhedrosis virus (EpNPV) have been registered as biopesticides in China and commercialized (Ye et al., 2014). However, viral biopesticides are currently used sparingly in tea plantations, primarily due to the narrow host ranges. Moreover, the term “virus” may negatively impact consumer and farmer perceptions.

### 2.2 Pathogen antagonistic microorganisms

In tea plantations, where fungal pathogens are the primary cause of diseases, research has predominantly focused on fungal antagonistic microorganisms. Antagonistic microorganisms inhibit pathogenic growth through mechanisms such as competition, siderophore production, antibiotic secretion, and quorum sensing (Fenta et al., 2023), with their effects often being complex and multifaceted. Some plant growth-promoting rhizobacteria (PGPR) not only exhibit growth-promoting characteristics but also possess antibacterial, insecticidal, and plant resistance-inducing properties (Khoso et al., 2024). In this context, they are discussed as MBCAs. For instance, *Serratia marcescens* ETR17, isolated from the rhizosphere of tea plants, demonstrated *in vitro* antagonistic activity against nine tea plant pathogens (Dhar Purkayastha et al., 2018). This antagonism was primarily achieved through the secretion of various hydrolytic enzymes (chitinase, protease, lipase, cellulase) and antibiotics (pyrrolnitrin and prodigiosin). Additionally, ETR17 also produced the plant hormone indole-3-acetic acid (IAA) and siderophore. This suggests that MBCAs may simultaneously play a role in both biocontrol and improving the quality of tea.

*Bacillus* species are known for their rapid growth, production of various antimicrobial metabolites and enzymes, and widespread application in plant disease management. Yuan et al (He et al., 2024). isolated *Bacillus velezensis* CSUFT-BV4 from healthy oil tea (*Camellia oleifera*) leaves, which exhibited up to 73.2% inhibition against five pathogens causing tea oil anthracnose. Among the Actinobacteria members, *Streptomyces* are recognized for their abilities to produce antifungal, insecticidal, and growth-promoting metabolites (Borah et al., 2022; Das et al., 2024). Marimuthu et al (Marimuthu et al., 2020). identified *Streptomyces* sp. SLR03 from river soil samples, which demonstrated effective antagonistic activity against the tea pathogen *Pestalotiopsis theae*. Studies have demonstrated that internal control mechanisms are more effective than external applications, highlighting endophytes as a valuable source of biocontrol agents. Thoudam et al (Thoudam and Dutta, 2012). evaluated the efficacy of several epiphytic fungi on tea plants for controlling *Corticium theae* Bernard, which causes black rot. Their findings indicated that *Aspergillus niger* exhibited the most effective inhibition, followed by *Trichoderma atroviride* and *Trichoderma cithnoviride*.

*Trichoderma* species are considered among the most promising antagonistic fungi (Thambugala et al., 2020). Research has demonstrated that *T.atroviride*, *T. asperellum*, and *T. harzianum* effectively control the wilt pathogen *Fusarium solani*, with inhibition rates ranging from 64.6% to 71.7%. Additionally, these fungi significantly promote the development of new shoots in tea plants (Kumhar et al., 2020). Kolandasamy et al (Kolandasamy et al., 2023). reported that *Bacillus subtilis VBS3* and *Trichoderma viride* VTV7, isolated from tea rhizospheres, effectively inhibited the mycelial growth and spore germination of *Phomopsis theae* by producing high levels of chitinase and β-1,3-glucanase. Shang et al (Shang et al., 2020). found that *T. asperellum* TC01 significantly reduced the severity of tea anthracnose caused by *Colletotrichum gloeosporioides*, with a reduction rate of 58.37%. Pandey et al (Pandey et al., 2022, 2023) observed that the strain *Trichoderma reesei* TRPATH01 exhibited antagonistic activities of 81.3% and 82.6% against the pathogens *Pseudopestalotiopsis theae* and *F.solani*, respectively on tea plants, through the production of inhibitory metabolites. Furthermore, Avascular Mycorrhizal Fungi (AMF) can also exhibit antagonistic activity. *Rhizophagus intraradices* BGC JX04B were able to reduce lesions caused by *Colletotrichum camelliae* in tea plants by 35.29% (Chen et al., 2022).

Fungal viruses have also shown potential in managing fungal diseases in tea plantations. For instance, Pestalotiopsis theae chrysovirus-1 (PtCV1), a fungal virus isolated from the tea pathogen *P.theae*, belongs to the family Chrysoviridae and the genus Alphachrysovirus. This virus significantly reduces the growth rate and virulence of its host fungus, effectively converting it into a non-pathogenic endophyte on tea leaves (Zhou et al., 2021). Furthermore, research indicates that plants can participate in the spread of fungal viruses (Hai et al., 2024). For example, after infection with *Sclerotinia sclerotiorum*, the concentration of proline in the plant significantly increases. This rise in proline weakens the fungus’s non-self-recognition response, thereby facilitating the spread of fungal viruses within the plant.

Despite the significant success of MBCAs in managing pests and diseases, their effectiveness still falls short compared to some chemical formulations. The efficacy of MBCAs in field conditions largely depends on factors such as their intrinsic activity, interactions with the natural microbiome, colonization ability within the ecosystem, and environmental adaptability (do Nascimento et al., 2022). Combining MBCAs with chemical treatments may offer effective pest and disease control while reducing the reliance on chemical agents (Jeyaraman and Robert, 2017). Additionally, the development of synthetic metal nanoparticles with biological activity represents an emerging and promising direction (Mythili Gnanamangai et al., 2017). However, it is essential to consider the broader ecological impacts of introducing exogenous microorganisms into tea plantations. Beyond their role in controlling pests and pathogens, studies have shown that some MBCAs can promote tea plant growth and enhance tea quality (Dhar Purkayastha et al., 2018; Bhattacharyya et al., 2020; Kumhar et al., 2020; Bora et al., 2022a; Pandey et al., 2022; He et al., 2024). Despite these findings, the underlying mechanisms remain largely unexplored. It is hypothesized that these effects may result from the complex interactions between the microorganisms and the tea plants. Therefore, further research is needed to elucidate the impact of MBCAs on the microecological dynamics of tea plantations and the metabolic processes of tea plants, ensuring a comprehensive understanding of their potential benefits and limitations.

## 3 Comprehensive impact of microbial biocontrol agents in tea plantations

### 3.1 Colonization efficacy of microbial biocontrol agents in tea plantations

Tea grower generally expect MBCAs to fulfill two key criteria: effective suppression of pathogens or pests and sustained survival in the environment. When conditions are favorable, MBCAs can be released as inoculants and remain in the microecosystem to prevent pest outbreaks or inhibit pathogen proliferation (McGuire and Northfield, 2020). One example is that MBCAs products containing *Pseudomonas fluorescens* in the U.S. often failed because these non-sporulating bacteria cannot survive long-term in natural environments (Choudhary and Johri, 2009). But studies have shown that sporulating Bt also exhibits poor colonization ability on plant leaves (Pedersen et al., 1995), likely due to its limited competitive ability in foliar environments (Maduell et al., 2008). The colonization of MBCAs on host plants is influenced by multiple factors, including inoculation methods, microbial species, crop types, growth conditions, and carriers (Bamisile et al., 2018), cannot be attributed to a single factor. Research on the colonization efficacy of microbial agents on tea plants is crucial for advancing microbial control strategies.

Nutritional supplements may aid in enhancing the survival of MBCAs under adverse conditions (Lin et al., 2023a). Certain strains adapted to tea plantation environments, such as *Streptomyces sannanensis*, are particularly suited for tea cultivation due to their ability to thrive in acidic soils (Gnanamangai and Ponmurugan, 2012). Furthermore, due to the saprophytic nature of *Trichoderma harzianum*, *Gliocladium virens*, *P.fluorescens*, and *Trichoderma atroviride*, they can occupy advantageous ecological niches in both soil and tea leaves, thereby altering the ecological functions of microbial communities (Sanjay et al., 2008; Manjukarunambika et al., 2013). These biocontrol agents also form biofilms and produce various cell wall-degrading enzymes upon colonizing host plants, thereby serving as effective barriers against pathogen invasion.

### 3.2 Impact on tea plant growth and tea quality

The ultimate goal of employing various management strategies in tea plantations is to enhance tea quality and mitigate adverse factors affecting. Key metabolic compounds in tea are critical indicators of quality. For instance, caffeine and catechins jointly influence the color and bitterness of tea. Non-protein amino acids, particularly theanine, are closely associated with the freshness and sweetness of tea. Volatile compounds such as linalool and cis-3-hexenol determine the aroma of tea (Zhang et al., 2020b). Stress from pests and pathogens, along with various exogenous factors, can affect the metabolic responses of tea plants, thereby altering the types and concentrations of metabolites in the tea leaves (Liao et al., 2019). Some MBCAs, in addition to their insecticidal and antimicrobial properties, have been shown to improve tea quality (Afridi et al., 2024). For example, growth-promoting effects associated with endophytic colonization by *B.bassiana* (Sánchez-Rodríguez et al., 2018) and *Metarhizium* species (Ahmad et al., 2020) have been observed in various plants. The growth-promoting mechanisms of MBCAs encompass the synthesis of phytohormones and siderophore, phosphate solubilization, potassium release, and nitrogen fixation (Barelli et al., 2020).

In tea plants, the antagonistic microorganism *Streptomyces sannanensis* has been shown to significantly improve both tea yield and quality parameters (Gnanamangai and Ponmurugan, 2012). Similarly, *Ochrobactrum anthropi* BMO‐111 has been reported to significantly increase the levels of chlorophyll, polyphenols, and catechins in tea buds. However, the observed increase in these indicators may be a result of the plant’s recovery from pathogen infection (Sowndhararajan et al., 2013). In another study, the rhizosphere strain *O. anthropi* TRS-2 demonstrated phosphate solubilization, iron carrier production, and IAA synthesis *in vitro*, confirming its plant growth-promoting mechanisms (Chakraborty et al., 2009). Huang et al (Huang et al., 2022). found that foliar application of *Bacillus amyloliquefaciens* to tea plants resulted in a decrease in the ratio of tea polyphenols to amino acids (TP/AA), catechin, and caffeine content, while the theanine content increased, with catechin reduction being associated with the biosynthesis pathway of flavonoids. Moreover, treatments with *T.asperellum* TC01 (Shang et al., 2020) and *T.reesei* TRPATH01 (Pandey et al., 2023) significantly enhanced parameters such as shoot height, stem diameter, weight of buds and roots in tea plants. These studies indicate that exogenous MBCAs may have a positive impact on tea quality. However, is this impact always beneficial? Although no direct reports have been found indicating adverse effects of MBCAs on tea plant growth, similar outcomes have been observed in other crops. For example, *Pseudomonas chlororaphis* IDV1 and *Pseudomonas putida* RA2, which antagonize the tomato pathogen *Ralstonia solanacearum*, exhibited good survival in maize rhizospheres but caused slight inhibition of maize growth (Kozdrój et al., 2004). Whether such effects arise from changes in the plant’s microecological structure, direct interference with plant metabolic processes, or other biological factors remains unclear.

### 3.3 Effects on non-target organisms and safety evaluation

As biological control agent use expands, it is essential to assess their potential impacts on non-target organisms in tea plantations. Non-target organisms, including non-tea plants, soil microbes, and wildlife. These organisms play significant roles within ecosystems, and disturbances to them could lead to ecological imbalances. Research has identified harmful effects of certain biocontrol agents on non-target species. For example, *B.bassiana* has been shown to have detrimental effects on some natural enemies (Li et al., 2024), while Bt exhibits toxicity to bees (Borges et al., 2021) and silkworms (Sandeep Kumar et al., 2016), which limits its application in sericulture countries such as India (Dashora et al., 2017). However, Bt proteins, particularly the ICPs, are highly specific and fully biodegradable, with no observed risk of toxic accumulation in the environment (Brar et al., 2007). Additionally, studies have indicated that injecting Bt crystal proteins into mice does not produce toxic effects (Román Calderón et al., 2007). Nevertheless, another study found that Bt inoculation might inhibit the root colonization of AMF by releasing suppressive compounds (Ferreira et al., 2003).

Plant-derived insecticides such as sophoridine and neem extract have not shown significant impacts on the populations of ladybugs, spiders, or parasitic wasps in tea plantations (Tian et al., 2020). However, neem extract has demonstrated adverse effects on the rhizosphere microbial communities of leguminous plants, resembling the effects of chemical pesticides (Singh et al., 2015a, 2015b). Notably, research has shown that bacterial inoculants can mitigate such negative impacts (Singh et al., 2022). Additionally, some Gram-negative bacteria used in biological control, such as *O. anthropi* strains, have potential as human pathogens. However, in murine models, the pathogenicity of *O. anthropi* strain BMO-111 was not observed, even with high inoculum concentrations (0.5 ml of 1 × 10^7^ CFU ml^−1^) orally administered to mice (Sowndhararajan et al., 2013).

## 4 Impact of microbial biocontrol methods on tea plant microbial communities

Microbial communities associated with tea plants can be broadly categorized into three main types: phyllosphere, rhizosphere, and endophytic microorganisms. Each of these groups plays a unique role in the plant’s microecology and overall health. Under-standing their characteristics and interactions is crucial for effectively applying microbial biocontrol methods to improve tea plant resilience and productivity.

### 4.1 Phyllosphere microorganisms

Phyllosphere microorganisms refer to the microbial communities associated with the aerial parts of plants (Xu et al., 2022a). The phyllosphere functions as an open system, making it susceptible to external factors such as ultraviolet radiation, air pollution, and microbial inoculation. Plant genotypes play a key role, shaping tissue structure and secondary metabolites (Ohler et al., 2022). In tea plants, the phyllosphere microbial community predominantly consists of *Proteobacteria*, *Actinobacteria*, *Bacteroidetes*, and *Firmicutes* at the phylum level. At the genus level, the core microbial taxa include bacteria such as *Herbaspirillum*, *Massilia*, *Methylobacterium*, *Pantoea*, *Pseudomonas*, and *Sphingomonas*. The diversity of phyllosphere fungi is generally lower than that of bacteria. These fungi can inhabit the phyllosphere in either epiphytic or endophytic forms, with predominant taxa including *Basidiomycota*, *Ascomycota*, and *Mortierella* (Xu et al., 2023b). Fungi and bacteria in the phyllosphere can collaborate in the metabolic processes of tea plants, with this interaction being more pronounced in young leaves (Lin et al., 2022).

The assembly of phyllosphere microbial communities and their interactions with host metabolites are crucial for the health of tea plants. Xu et al (Xu et al., 2022b). demonstrated that the phyllosphere microbiota varies with the metabolic products of tea leaves at different developmental stages, although a stable core microbial community exhibits antagonistic effects against various pathogens (Xu et al., 2022b). Microorganisms such as *Sphingomonas*, *Herbaspirillum*, and *Massilia* can migrate from the soil to the leaves and maintain phyllosphere homeostasis (Xu et al., 2023b). Additionally, Bt is also a common component of many plant phyllosphere microbiota, where it can interact with and kill herbivorous insect larvae (Uribe et al., 2003; Collier et al., 2005). Key drivers of microbial community assembly in tea plants include metabolites like caffeine and epigallocatechin gallate (Xu et al., 2022b), suggesting that tea plants may recruit beneficial microorganisms by secreting specific metabolites under biotic stress. Similarly, Xie et al (Xie et al., 2022). found that tea plants influence rhizosphere bacterial diversity, community structure, and nitrogen cycling-related gene abundance through the secretion of L-theanine into the rhizosphere. This indicates the presence of complex interactions among insects, microorganisms, and tea plants.

The use of chemical agents in agriculture can disrupt phyllosphere microbiota (Chen et al., 2021). For example, a study on wheat found that while the herbicide S-metolachlor did not significantly affect plant physiology, it reduced the diversity of the phyllosphere microbiota, indicating that microbial communities are sensitive indicators of short-term plant stress (Xu et al., 2020). MBCAs have shown significant impacts on the microbiota of various crops (Zhang et al., 2008; Sylla et al., 2013; Perazzolli et al., 2014; Wang et al., 2014; Qin et al., 2019; Hao et al., 2021), though some studies have reached the opposite conclusion (Wei et al., 2016). For instance, Bt does not significantly alter the bacterial community on the leaves of cabbage (*Brassica oleracea*) (Russell et al., 1999) or the phyllosphere bacterial community of *Oryza sativa* (Wang et al., 2014), likely due to Bt’s weak competitive ability (Maduell et al., 2008). Evidence also suggests that combined microbial inoculants have a more pronounced effect on microbial community structure compared to single-agent applications (Xu and Jeger, 2013), and repeated application of MBCAs may increase opportunities for establishing active populations (Wei et al., 2016). However, microbial combinations can lead to competitive or antagonistic relationships, necessitating careful consideration. While research on MBCAs in tea plant phyllosphere microbiota is limited, studies on other crops indicate that MBCAs may influence tea plants. Further research is needed to understand the specific mechanisms by which MBCAs affect tea plant phyllosphere microbiota.

### 4.2 Rhizosphere microorganisms

In the rhizosphere of tea plants, AMF and various other microorganisms play key roles in processes such as phosphate solubilization, nitrogen fixation, iron chelation, stress tolerance, and auxin production (Chen et al., 2023). In tea plantations, soil fungi predominantly belong to *Ascomycota*, *Mortierellomycota*, and *Basidiomycota*, while the dominant bacterial phyla are *Acidobacteria*, *Actinobacteria*, and *Proteobacteria* (Jibola-Shittu et al., 2024). Research suggests that more complex soil ecological networks may contribute to the suppression of tobacco wilt disease (Yang et al., 2017). Additionally, rhizosphere bacteria from tea plantations, such as *Burkholderia* sp. AULS-B3, has been found to both metabolize the pesticide endosulfan and promote tea plant growth (Huidrom and Sharma, 2024).

The stability of tea plant rhizosphere microbial communities is shaped by agronomic practices (Zou et al., 2022; Zhang et al., 2023). Lin et al (Tan et al., 2019). observed that organic tea plantations (OTP) exhibit significantly higher alpha diversity and Chao1 indices than conventional tea plantations (CTP) (Sang and Kim, 2012). Exogenous microbial applications can also influence native rhizosphere microbiome, though the effects may be ambiguous. For instance, lower concentrations of *Beauveria bassiana* spore suspensions improved paddy soil yields by affecting microbial community structure and enzyme activity (Du et al., 2013). In soybeans (*Glycine max*), Bt did not alter cultivable heterotrophic bacteria or saprophytic fungi in the rhizosphere (Ferreira et al., 2003). Similarly, applying *Pseudomonas fluorescens* DR54 to barley (*Hordeum vulgare*) temporarily shifted the rhizosphere microbiome (Johansen and Olsson, 2005) due to competition with native microbes, causing DR54 decline. This phenomenon explains why most MBCAs rapidly decline in number after soil introduction, and microbial communities tend to recover quickly.

The relationship between rhizosphere microorganisms and their host plants is highly intricate. For instance, the soil-dwelling insect-pathogenic fungus *Metarhizium robertsii* possesses two adhesin genes: MAD1, which facilitates attachment to the insect cuticle, and MAD2, which aids in adhesion to plants, highlighting its association with plant hosts (Sasan and Bidochka, 2012). Similar to the phyllosphere, plant roots release signaling molecules into the soil under stress, recruiting beneficial microorganisms to alleviate various environmental pressures. These signaling molecules are likely plant hormones or other secondary metabolites. For example, research by Berendsen et al (Berendsen et al., 2018). showed that plants can attract beneficial microorganisms by regulating hormone levels when infected by pathogens. Studies suggest that pathogen-infected roots secrete more amino acids, nucleic acids, and long-chain organic acids, which alter the root microbiome and enhance disease resistance (Yuan et al., 2018). The application of MBCAs can also change plant hormone levels, indicating that these microbial shifts are the result of multiple interacting factors, often regulated by the plant itself. This underscores the mutualistic relationship between plants and microorganisms, suggesting that selecting beneficial MBCAs could promote an ideal symbiosis.

### 4.3 Endophytic microorganisms

Endophytic microorganisms, which reside within plant tissues, have a particularly close relationship with their host plants (Wang and Cernava, 2020). Research highlights their critical role in promoting plant growth and health (Bastias et al., 2017). For instance, endophytic bacteria can enhance plant resistance to herbivorous insects through jasmonic acid (JA)-mediated defense pathways (Bastias et al., 2017). Most isolated endophytes, primarily from the *Bacillus* genus, exhibit plant growth-promoting abilities. However, the evaluation of MBCAs often neglects the impact on endophytes (Kabir et al., 2023). This omission may be due to the cryptic endophytic environment, the diversity and heterogeneous distribution of endophytes, which pose significant research challenges. Because endophytes are frequently grouped together with phyllosphere microorganisms in studies. While this simplifies experimental design, it overlooks the unique functions of endophytes. Future research should emphasize the interactions between tea plant microbiota and MBCAs, particularly in diverse ecological systems and management practices. Understanding how these interactions alter microbial communities is essential for developing beneficial microecologies that enhance tea plant resistance and quality.

## 5 Insects, microorganisms, and tea plant metabolism

**Figure 2:**
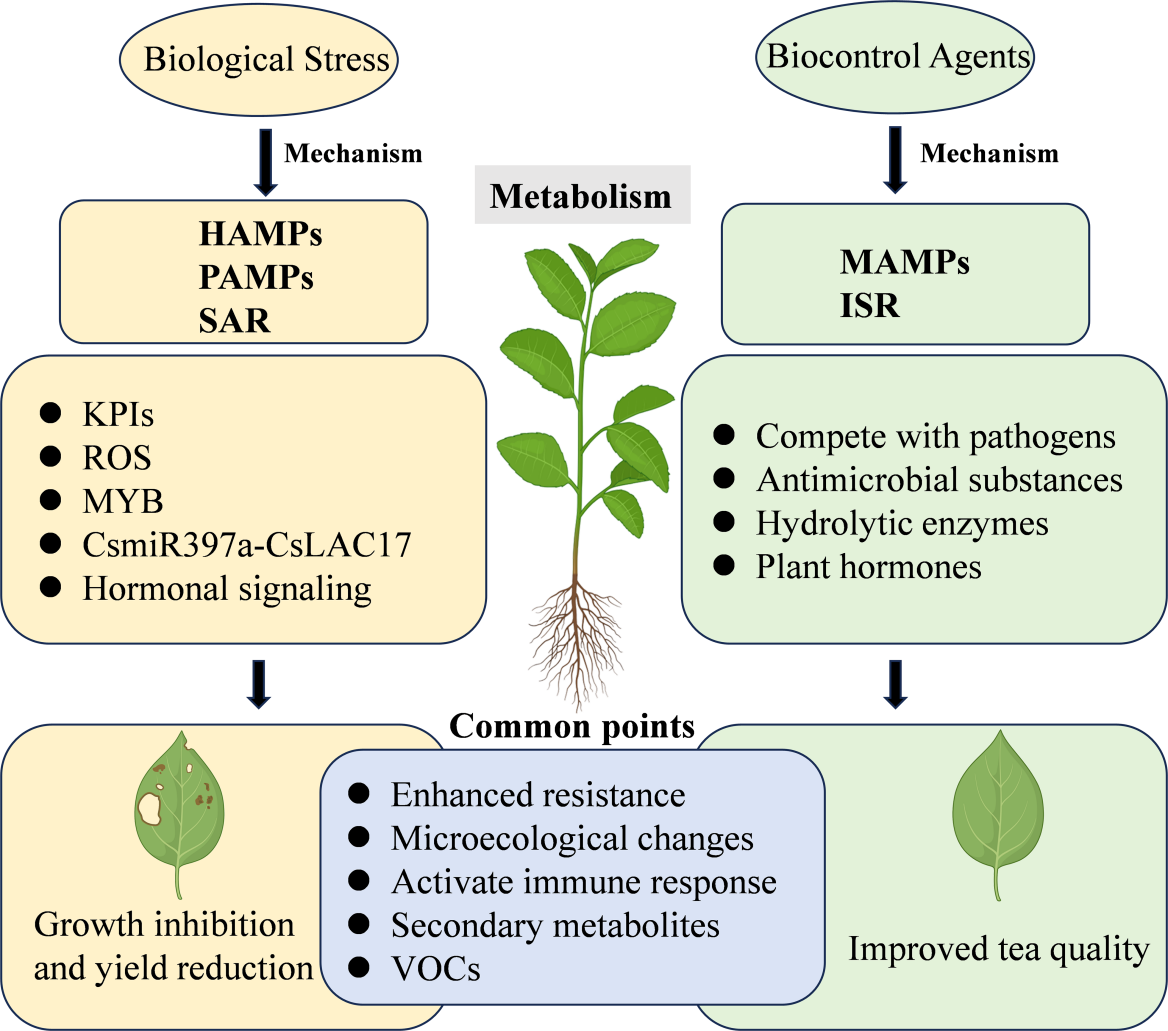
The respective impacts of MBCAs and biological stress factors on the metabolic mechanisms of tea plants. HAMPs, herbivore-associated molecular patterns; PAMPs, pathogen-associated molecular patterns; MAMPs, microbe-associated molecular patterns; SAR, systemic acquired resistance; ISR, induced systemic resistance; KPIs, proteinase inhibitors; ROS, reactive oxygen species; MYB, MYB transcription factors; VOCs, volatile organic compounds.

The intricate interplay between insects, microorganisms, and tea plant metabolism significantly influences the overall health and productivity of tea plants. Understanding tea plant metabolic responses to pest and disease stress is crucial for developing effective management strategies. Additionally, MBCAs not only control pests and diseases but also influence tea plant metabolism, enhancing resilience and quality. This section examines into the response mechanisms of tea plant metabolism under stress and explores the role of MBCAs in mediating these metabolic processes (**Figure 2**).

### 5.1 Response mechanisms of tea plant metabolism under pest and disease stress

Tea plants have evolved complex metabolic pathways and defense mechanisms. Pathogen attacks trigger systemic acquired resistance (SAR) in uninfected tissues, providing broad-spectrum resistance (Jeyaraj et al., 2017; Hilleary and Gilroy, 2018; Naskar et al., 2021; Zhang et al., 2024). Local responses triggered by herbivore-associated molecular patterns (HAMPs) and pathogen-associated molecular patterns (PAMPs) are rapid and intense, while systemic responses involve secondary metabolites, proteinase inhibitors (KPIs), reactive oxygen species (ROS), and plant hormones to combat further assaults (Fu and Dong, 2013; Naskar et al., 2021; Jomova et al., 2024). Under stress from pests and pathogens, tea plants also exhibit significant changes in primary metabolic pathways. Primary metabolites such as carbohydrates, amino acids, and organic acids are not only fundamental to tea plant growth and development but also play crucial roles in its defense mechanisms (Zhou et al., 2015).

For example, tea looper (*Ectropis obliqua*) feeding induces systemic carbon and nitrogen redistribution to bolster secondary metabolism for defense (Huang et al., 2018; Yang et al., 2019; Li et al., 2020). During this process, L-theanine is primarily synthesized by CsTSI in the roots and transported through the vascular system to new tea shoots (Lin et al., 2023b), while caffeine (1,3,7-trimethylxanthine) is predominantly synthesized in chloroplasts (Xia et al., 2017; Liao et al., 2022). This coordinated metabolic reorganization helps tea plants maintain a balance between growth and defense when facing external pressures. RNA sequencing (RNA-Seq) analysis has revealed 1,859 differentially expressed genes (949 upregulated and 910 downregulated) in tea plants subjected to *E. oblique* infestation compared to controls. These genes are involved in signal transduction, insect defense response transcription factors, phenylpropanoid pathways, herbivory-induced plant volatiles (HIPVs), and caffeine biosynthesis (Wang et al., 2016b). Similarly, *Exobasidium vexans* infection revealed 149 defense-related genes in resistant tea genotypes, including defense-related enzymes, resistance genes, multidrug-resistant transporters, transcription factors, retrotransposons, metacaspases, and chaperones (Jayaswall et al., 2016). Beyond local responses, tea plants also activate defense-related genes in adjacent leaves through signaling mechanisms (Zhou et al., 2020).

Plant hormones play a crucial regulatory role in the defense responses of tea plants (Liao et al., 2019, 2022; Yang et al., 2019). Jasmonic acid (JA) and salicylic acid (SA) are key signaling molecules that activate defense pathways (Zhang et al., 2020a; Hou and Tsuda, 2022), JA is primarily involved in tea plant responses to folivorous insects, while SA predominantly mediates immune responses to microbial pathogens (Zhu et al., 2019; Hu et al., 2022; Sultana et al., 2024). Studies have shown that JA and SA play important roles in shaping the root microbiome (Lebeis et al., 2015). Additionally, other plant hormones such as ethylene (ET), abscisic acid (ABA), auxin (indole-3-acetic acid, IAA), cytokinins (CTK), and gibberellins (GA) are required to ensure proper coordination between growth and defense (Bigeard et al., 2015; Ye et al., 2021). Although JA and SA are generally considered antagonistic (Jiao et al., 2022), studies have shown that they can also act synergistically (Liu et al., 2016). For instance, attacks by both piercing-sucking insects like the tea green leafhopper (*Empoasca onukii*) and chewing insects like tea looper (*Ectropis grisescens*) lead to increased levels of both JA and SA (Jin et al., 2020). Notably, *E. onukii* attack also raises ABA levels, which may relate to the insect’s mode of injury (Liao et al., 2019). Conversely, simulated attacks by piercing-sucking insects alone elevate JA and ABA levels in tea leaves, with no significant change in SA levels. This raises the question of whether SA variation might be related to insect salivary effectors or microbial interactions (Xu et al., 2019; Smets and Koskella, 2020). Furthermore, Zhao et al (Zhao et al., 2020b). found that invasion by *E. onukii* upregulated genes related to the biosynthesis of phenylpropanoids and flavonoids and induced the synthesis of cuticular waxes, leading to increased levels of C29 alkanes. Tea plants infected with *Pseudopestalotiopsis camelliae-sinensis* may enhance lignin content in young tea shoots through the CsmiR397a-CsLAC17 module, thereby reducing stem tenderness and increasing resistance to tea leaf spot disease (Yang et al., 2024). The dynamic adjustment of these metabolic pathways not only enhances tea plant survival but also impacts the flavor and aroma of the tea.

As previously discussed, characteristic metabolites in tea plants, such as terpenoids, phenolic compounds (flavonoids, anthocyanins, lignins, and tannins), and nitrogen-containing compounds (such as alkaloids and non-protein amino acids), not only protect the tea plant but also determine the overall flavor and health benefits of tea (Hilleary and Gilroy, 2018; Yu and Yang, 2020; Zhao et al., 2020a, 2023b; Zeng et al., 2021; Li et al., 2022). It has been demonstrated that catechins and caffeine can inhibit pathogen growth *in vitro* (Bansal et al., 2012). For instance, infestations by *E.oblique* and *Colletotrichum fructicola* increase the biosynthesis of flavonoids and caffeine in tea leaves, accompanied by elevated expression of related synthesis genes (Wang et al., 2016a). Interestingly, certain *Pseudomonas* species in the gut microbiota of the coffee borer beetle (*Hypothenemus hampei*) use caffeine as their sole carbon and nitrogen source (Ceja-Navarro et al., 2015), suggesting that some insects may have evolved adaptive detoxification systems for caffeine, which could be relevant for research on tea plants as well. Additionally, changes in metabolite levels evidently influence microbial communities. For example, after infection with *E.vexans*, the dominant endophytic fungal community in tea plants shifted from *Ascomycota* to *Basidiomycota*, while the relative abundance of *Actinobacteria* in bacteria increased (Cao et al., 2023).

Tea plant volatile organic compounds (VOCs) play a dual role in both aromatic and defensive functions (Zeng et al., 2019b). They can repel pests (Jing et al., 2021), attract natural enemies (Zeng et al., 2019b), and trigger immune responses in neighboring plants (Bouwmeester et al., 2019). For instance, after feeding by *E.oblique*, tea plants release compounds such as S-linalool and β-ocimene, which significantly attract the parasitic wasp *Parapanteles hyposidrae* (Liu et al., 2024a). Due to the aromatic properties of VOCs, the impact of pest infestations on tea quality is not always negative. For example, feeding by the green leafhopper (*Jacobiasca formosana*) increases the release of (S)-linalool and geraniol, and induces the production of diene alcohol I. This process contributes to the Oriental Beauty Oolong tea, which is known for its distinctive aroma (Cho et al., 2007).

Overall, tea plants exhibit a sophisticated metabolic regulatory system in response to pest and pathogen pressures. These mechanisms encompass not only the accumulation of plant hormones and secondary metabolites but also the regulation of carbon and nitrogen metabolism, the generation and scavenging of reactive oxygen species, the release of volatile organic compounds, and systemic defense responses in adjacent leaves. These insights offer new possibilities for pest and disease management in tea cultivation. For example, exogenous application of JA and SA can enhance both the quantity and quality of volatile compounds (Chen et al., 2020). Additionally, inducing tea plant disease resistance genes through external agents may improve the plant’s disease resistance (Senthilkumar et al., 2012; Lu et al., 2019). Future research could further elucidate the specific regulatory mechanisms of these metabolic pathways, potentially providing novel strategies for genetic improvement and integrated pest and disease management in tea cultivation.

### 5.2 Microbial biocontrol agents mediating tea plant metabolism

In tea plants, inoculation with weak pathogens or non-pathogenic microbial agents can trigger an enhanced resistance state known as induced systemic resistance (ISR) (Pieterse et al., 2014). Typically, plant immune responses are triggered by microbe-associated molecular patterns (MAMPs), leading to microbe-associated molecular pattern-triggered immunity (MTI). MTI functions by recognizing pathogen-associated molecular patterns and activating defense signaling pathways. However, this defense response often leads to a reallocation of resources, consequently inhibiting plant growth (Shang et al., 2020; Ma et al., 2021). Unlike MTI, ISR regulates the expression of defense genes through the jasmonic acid (JA) and ethylene signaling pathways, promoting the accumulation of defense metabolites and protecting distal tissues. While both MTI and ISR enhance disease resistance, ISR also has the potential to promote plant growth, providing additional growth advantages alongside conventional immune defenses via MTI (Kusajima et al., 2018; Yu et al., 2022). However, this advantage may come with potential trade-offs due to differences in resource allocation.

In studies conducted in India, the application of *Pseudomonas fluorescens* and *Pseudomonas aeruginosa* strains RRLJ 134 and RRLJ 04, respectively, has been shown to activate systemic resistance in tea plants against *Fomes lamoensis* and *Ustulina zonata* (Mishra et al., 2014). These treatments, along with antagonistic microorganisms *Bacillus altitudinis* GS-16 and *T. reesei* TRPATH01, significantly enhanced key defense enzyme activities in tea plants (Pandey et al., 2022; Wu et al., 2024b). Additionally, antagonistic fungi *Trichoderma asperellum* TC01 treatment activated the expression of genes associated with flavonoids, phenylpropanoids, jasmonic acid, and ethylene (Shang et al., 2020). Treatment with the AMF *Rhizophagus intraradices* BGC JX04B resulted in a significant increase in superoxide anion levels, as well as the activities of catalase and peroxidase in young tea seedlings. This response is likely mediated through pathways involved in plant hormone signaling, mitogen-activated protein kinase (MAPK) signaling, and phenylpropanoid biosynthesis (Chen et al., 2022).

In other crops, a method analogous to the use of “inactivated vaccines” in medicine involves the application of fungal pathogen *Stemphylium lycopersici* mycelial suspensions, which have been attenuated by infection with Stemphylium lycopersici Alternavirus 1 (SlAV1), to enhance plant resistance against virulent strains and convert pathogenic fungi into biocontrol agents (Liu et al., 2022). Overall, MBCAs have a significant impact on the metabolism of tea plants, with some changes in metabolic pathways resembling the plant’s response to pathogen and pest stress. As shown in **Table 3**, both stress and the application of MBCAs can enhance the resistance of tea plants.

**Table 3:** The impact of exogenous biological factors on tea tree metabolism.

Biological Factors	The Impact on Tea Tree Metabolism
*Ectropis oblique*	Signal transduction, Carbon and nitrogen resource allocation, Phenylpropanoid biosynthesis; Flavonoids and caffeine biosynthesis, Herbivory-induced plant volatiles (HIPVs) (Wang et al., 2016b; Yang et al., 2019; Li et al., 2020)
*Empoasca onukii*	Plant hormones (JA, SA and ABA), Phenylpropanoid and cuticular wax biosynthesis, Flavonoids biosynthesis, (S)-Linalool, Geraniol, Diene diol I (Cho et al., 2007; Jin et al., 2020; Zhao et al., 2020b)
*Ectropis grisescens*	Plant hormones (JA, ethylene and auxin), Three catechin compounds (epicatechin, [+]-catechin and epigallocatechin) (Li et al., 2022)
*Exobasidium vexans*	Defense related enzymes, Resistance genes, Multidrug resistant transporters, Transcription factors, Retrotransposons, Metacaspases and chaperons, Dominant microorganisms change (Jayaswall et al., 2016; Cao et al., 2023)
*Colletotrichum fructicola*	S-Adenosylmethionine Synthetase (SAMS), Tea Caffeine Synthase1 (TCS1) 和Leucoanthocyanidin Reductase (LAR) (Wang et al., 2016a)
*Pseudopestalotiopsis camelliae-sinensis*	CsmiR397a-CsLAC17 module (Yang et al., 2024)
*Toxoptera aurantii* Boyer	Flavonoids biosynthesis, Plant hormones (JA) (Liu et al., 2024b)
*Pseudomonas* sp.	Defense related enzymes (L-phenylalanine ammonia-lyase, Peroxidase, Polyphenol oxidase) (Mishra et al., 2014)
*Bacillus megaterium*	Defense related enzymes (Peroxidase, Chitinase, β-1,3-glucanase, Phenylalanine ammonia-lyase) (Chakraborty et al., 2006)
*Bacillus altitudinis*	Defense related enzymes (Polyphenol oxidase, Superoxide dismutase, Phenylalanine ammonia-Lyase) (Wu et al., 2024b)
*Trichoderma Asperellum*	Plant hormones (JA and ET), Flavonoids and phenylpropanoid biosynthesis (Shang et al., 2020)
*Trichoderma reesei*	Defense related enzymes (Polyphenol oxidase, Peroxidase, Phenylalanine ammonia-lyase, Phenolic compounds, β-1,3-glucanase, Chitinase) (Pandey et al., 2022)
*Rhizophagus intraradices*	Plant hormones, Mitogen-Activated Protein Kinase (MAPK), Signal transduction, Phenylpropanoid biosynthesis (Chen et al., 2022)

## 6 Conclusions and future perspectives

This review primarily summarizes the potential of microbial biocontrol agents (MBCAs) in enhancing both the management of tea tree diseases and the quality of tea leaves. The use of MBCAs represents a shift from merely targeting and killing pests or pathogens to protecting tea trees and improving their overall health. Integrating MBCAs into tea plantation management practices can bolster the natural defenses of tea trees and improve the overall quality of tea by fostering beneficial microbial communities. This approach also provides a valuable model for the application of microbial biocontrol agents in other perennial crop systems. However, existing research faces challenges such as limited effectiveness, stability issues, high costs, and gaps in understanding the molecular mechanisms of MBCAs. Future research directions should focus on:

1. Development and Application of Novel Microbial Biocontrol Agents: Future studies should aim to discover and develop more effective MBCAs, especially those targeting specific diseases or pests affecting tea trees. Additionally, research should explore the integration of MBCAs with tea plantation management practices such as fertilization, irrigation, and crop rotation. Optimizing these integrated management strategies could lead to a comprehensive microbial biocontrol approach for tea plantation management.
2. Mechanisms of MBCAs on Tea Quality: Understanding how MBCAs improve tea quality is essential. Research should focus on how these agents influence the metabolic pathways of tea trees, especially in terms of nutrient uptake, stress responses, and the production of secondary metabolites that contribute to tea flavor and aroma. Investigating how MBCAs improve tea quality by modulating the metabolic pathways of tea trees and promoting the establishment of beneficial microbial communities is essential. Understanding these mechanisms will help establish the relationship between tea tree microecology and tea quality, providing a scientific basis for optimizing ecological pest control in tea plantations and enhancing tea quality.
3. Addressing Limitations and Enhancing Efficiency of MBCAs: Current challenges with MBCAs include issues related to their stability, limited range of effectiveness, and high costs. Future research could focus on developing new carriers to maintain microbial activity, combining MBCAs with other control methods to enhance efficacy, and optimizing fermentation processes to reduce production and application costs. These efforts aim to make the use of MBCAs in tea plantation management more economically viable and effective.
